# Can Adding BMP2 Improve Outcomes in Patients Undergoing the SUPERhip Procedure?

**DOI:** 10.3390/children8060495

**Published:** 2021-06-10

**Authors:** Dror Paley, Claire E. Shannon, Monica Nogueira, Catharina Chiari, Matthew Harris

**Affiliations:** 1 Paley Orthopedic and Spine Institute, St. Mary’s Hospital, West Palm Beach, FL 33407, USA; cshannon@paleyinstitute.org; 2 Pediatric Orthopaedic Group, Orthopaedics Department, State Hospital of Sao Paulo, São Paulo 04024-002, Brazil; monipn@uol.com.br; 3 Department of Orthopedics and Trauma Surgery, Medical University of Vienna, 1090 Vienna, Austria; catharina.chiari@med.uniwien.ac.at; 4 Joint Preservation and Limb Reconstruction Center, Jupiter, FL 33477, USA; matt.harris735@gmail.com

**Keywords:** congenital femoral deficiency (CFD), proximal focal femoral deficiency (PFFD), coxa vara, bone morphogenic protein 2 (BMP2), endochondral ossification

## Abstract

Congenital femoral deficiency (CFD) Paley type 1b is characterized by severe bony deformity of the upper femur, extra-articular contractures of the hip, and_,_ delayed ossification of the femoral neck and/or subtrochanteric region. The Systematic Utilitarian Procedure for Extremity Reconstruction of the hip (SUPERhip) procedure for the correction of CFD deformities was developed in 1997. Initially, a non-fixed angle device (rush rod) was used for fixation. Late complications of persistent delayed ossification and recurrent varus deformity occurred. In order to reduce and treat such complications, fixation with a fixed angle device and the off-label use of BMP2 to induce ossification of the un-ossified femoral neck were employed. The purpose of this study is to determine if the use of a fixed angle device, and, BMP2 inserted into a drill hole in the cartilage of the femoral neck, decreases the incidence of these late complications. We retrospectively reviewed 72 SUPERhip procedures performed for Paley type 1b CFD between 1997 and 2012. Due to recurrent varus or persistent delayed ossification of the femoral neck, 34 revision SUPERhip procedures were performed. In total, 106 SUPERhip procedures were studied. Sixty-eight SUPERhips were performed using internal fixation without BMP2, while 38 SUPERhips were performed with both internal fixation and the addition of BMP2. Forty-one were performed using non-fixed angle internal fixation while 65 had fixed angle internal fixation. Fixed angle devices significantly reduced the incidence of recurrent varus compared with non-fixed angle devices. Inserting BMP2 in the femoral neck significantly reduced the incidence of persistent delayed ossification. Using only a fixed angle device but no BMP2 did not reduce the incidence of delayed ossification. The combination of both a fixed angle device and BMP2 reduced the incidence of recurrent coxa vara and persistent delayed ossification of the femoral neck. The SUPERhip procedure corrects the pathoanatomy of the proximal femur in CFD Paley type 1b but is associated with a very high risk of recurrence of coxa vara and persistence of femoral neck delayed ossification, unless, a fixed angle internal fixation device is used to prevent recurrent coxa vara and BMP2 is used to induce ossification of the femoral neck.

## 1. Introduction

**Figure 1:**
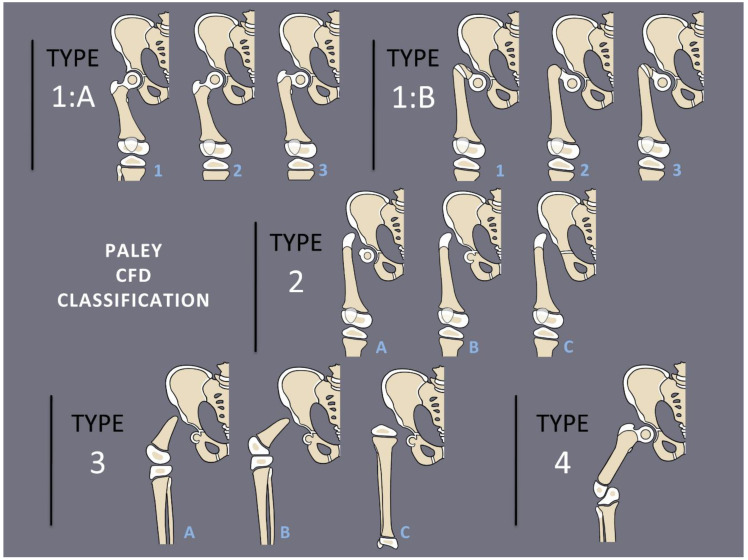
Paley Classification of Congenital Femoral Deficiency. Type 1A demonstrates normal ossification of the femoral neck and subtrochanteric region with normal version (1A_1_), retroversion (1A_2_), or varus and retroversion (1A_3_). Type 1B demonstrates delayed ossification of the subtrochanteric region (1B_1_), femoral neck (1B_2_), or combined type (1B_3_). Type 2 demonstrates an absence of the femoral neck with a mobile (2A), fused (2B), or absent (2C) femoral head. Type 3 demonstrates absence of the proximal femur with >45 degrees of knee motion (3A), <45 degrees of knee motion (3B), or complete femoral absence (3C). Type 4 demonstrates absence of the distal femur.

**Figure 2:**
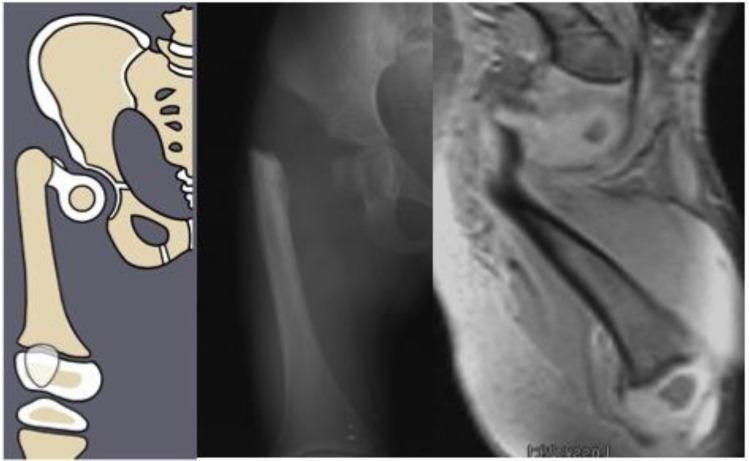
Paley type 1b_2_ (neck type) CFD example. Illustration (**left**), radiograph showing delayed ossification of femoral neck (**center**), and MRI showing cartilaginous neck (**right**).

Congenital femoral deficiency (CFD) is a spectrum of deformity, deficiency, and discrepancy of the femur, hip, and pelvis [1]. The most common presentation and most reconstructable types have bony and/or cartilaginous continuity between the femoral head and femoral diaphysis. These are classified using the Paley classification (Figure 1) as Paley type 1a (normal ossification) and type 1b (delayed ossification) (Figure 2) [1,2,3,4,5,6,7,8,9,10,11]. Type 1b cases have severe proximal femoral varus, flexion and retroversion, and acetabular dysplasia, as well as soft tissue flexion, external rotation, and abduction contractures of the hip [1,2,3,4,5,6,7,8,9,10,11].

The Systematic Utilitarian Procedure for Extremity Reconstruction of the hip, or SUPERhip procedure (SH), was developed by Paley in 1997 to address the soft tissue contractures and bony deformities of the hip that are present in CFD [1,2,3,4,5,6,7,8,9,10,11]. The characteristic flexion contracture of the hip is treated by performing multiple extra-articular soft tissue releases, including release of the tensor fascia lata and lengthening of the rectus femoris and psoas tendons. The abduction contracture is treated with relative lengthening of the abductor muscles by shortening the ilium, referred to as an abductor slide. The external rotation contracture is corrected by lengthening of the piriformis tendon. The severe bony varus, flexion, and retroversion of the proximal femur is corrected by performing a subtrochanteric realignment osteotomy with de-rotation and shortening of the femur. Finally, the acetabular dysplasia is addressed by performing pelvic osteotomy, such as the unicortical iliac osteotomy (previously referred to as the modified Dega) or a triple pelvic osteotomy using the lateral approach, as developed by Paley and described by Grigoryan et al. [12].

**Figure 3:**
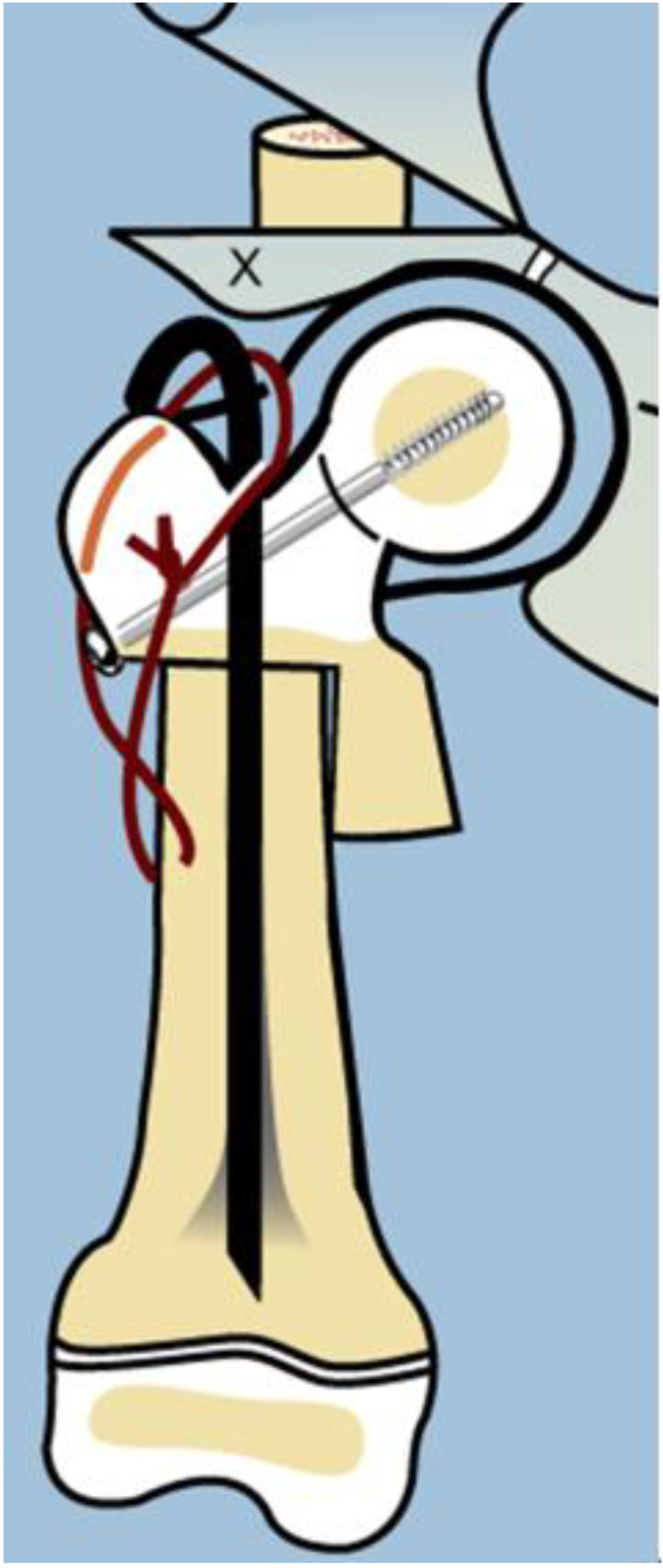
Illustration after SUPERhip procedure using a non-fixed angle device for fixation (Rush rod with tension band wire) and the neck was reinforced with a non-fixed angle screw.

As with many surgical techniques, there is an evolution process that is driven by trial and error. The SH procedure is no exception. In the original version of the SH, the proximal femur osteotomy was fixed with a rush rod and tension band wire construct (Figure 3) [7,8].

**Figure 4:**
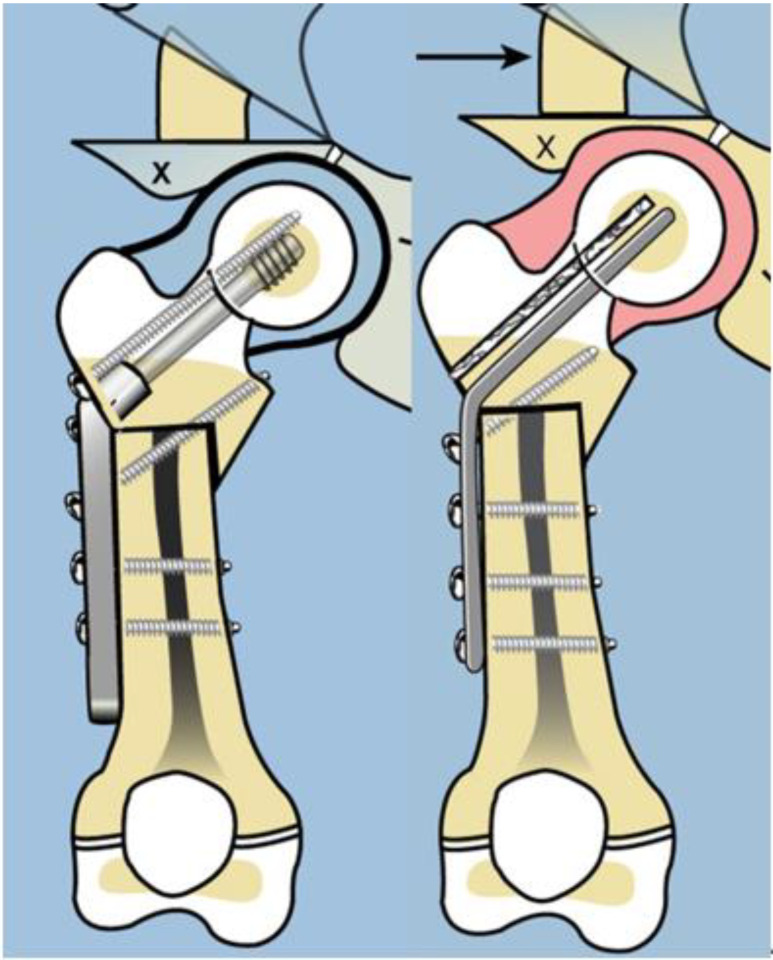
Illustration after SUPERhip procedure using a fixed angle device for fixation; sliding hip screw with additional rotation control second screw (left), and blade plate (right). Note on the right side there is a drill hole made for insertion of BMP2 superior to the blade.

Late complications, such as recurrent varus deformity and delayed ossification of the femoral neck, were reported in the earliest versions of the SH surgery [7,8]. In order to address the recurrent varus, the non-fixed angle fixation (Rush rod and tension band wire) was replaced with a much stronger fixed angle construct; a sliding hip screw (SHS) (Smith and Nephew, Memphis, TN, USA) or a 130 degree cannulated blade plate (Smith and Nephew, Memphis, TN, USA) [7,8] (Figure 4).

It was observed that many patients who developed recurrent varus also had persistent delayed ossification of the femoral neck. Bone morphogenic protein 2 (BMP2) (Infuse, Medtronic, Memphis, TN, USA), which works early in the bone formation pathway by inducing cartilage to turn into bone via endochondral ossification [13,14], was shown to be safe for use in humans without significant risk of oncogenesis [15]. BMP2 was adopted into the SH procedure to address persistent delayed ossification. BMP2 is not FDA cleared for use in children since no application for such clearance was ever made. Despite this, it has been used off-label in children for many years. The senior author has used BMP7 and BMP2 in children for approximately 20 years for various indications [16]. The BMP2 was inserted into the cartilage of the non-ossified femoral neck to induce conversion of the cartilage anlage into bone. The subjective impression of switching to a fixed angle device and using BMP2 improved the outcomes of the SH procedure and have become staples of the current technique. The purpose of this study is to objectively determine whether: (1) the use of a fixed angle device prevents recurrent coxa vara, and (2) BMP2 inserted into the femoral neck decreases the incidence of post-operative delayed ossification. 

## 2. Materials and Methods

Institutional review board approval was obtained for a retrospective review of all charts and radiographs for patients who underwent the SH procedure for CFD, performed by the senior author (D.P.), between 1997 and 2012. During this fifteen-year period, there were a total of 122 primary SH surgeries performed in 121 patients, and 36 revision SH surgeries performed in 24 patients. Each hip was classified pre-operatively according to the Paley classification for CFD. In the primary SH cohort, 26 hips were Paley type 1a (normal ossification), 24 were type 1b_1_ (subtrochanteric), and 72 were either type 1b_2_ (neck) or type 1b_3_ (combined). Of the 36 revision SH procedures, 2 were for type 1b_1_ hips, and 34 were for type 1b_2_ and 1b_3_ hips All revision procedures were performed for recurrent varus of the proximal femur or persistent delayed ossification of the femoral neck. The Paley type 1b_2_ and 1b_3_ hips were combined and are referred to hereafter as the “neck” group or type 1b_2_. All patients with Paley type 1a or 1b_1_ hips were excluded. This left a total of 72 primary SH procedures in 72 patients, all with type 1b_2._ There were also 34 revision SH procedures performed in 26 patients (8 patients required two revision SH procedures). For the purpose of this study, each revision SH was looked upon as an independent SH procedure. The following presentation of materials, results and analysis concern the 72 primary and 34 revision SH procedures, i.e., the 106 total SH procedures. 

The method of fixation used in all type 1b_2_ SH procedures prior to 2002 was a non-fixed angle construct, utilizing a Rush rod (Zimmer, Warsaw, IN, USA) and a tension band wire (Figure 3). After 2002, the method of internal fixation was changed to a fixed-angle device. Initially, this was a pediatric sliding hip screw (SHS) (Smith and Nephew, Memphis, TN, USA) and later, a 130° pediatric cannulated blade plate (BP) (Smith and Nephew, Memphis, TN, USA) (Figure 4). A non-fixed angle construct was used in 34/72 primary SH procedures and 7/34 revision SH procedures. A fixed angle device was used in 38/72 primary SH procedures, and 27/34 revision SH procedures_._ BMP2 (Infuse, Medtronic, Memphis, TN, USA) was inserted into the non-ossified portion of the femoral neck in 27/72 primary SH procedures. All 27 of these hips were treated with a fixed angle device. In the type 1b_2_ revision SH procedures, 11/34 were treated with BMP2 for persistent delayed ossification. None of the hips treated with revision SH for delayed ossification had undergone prior insertion of BMP2. 

**Figure 5:**
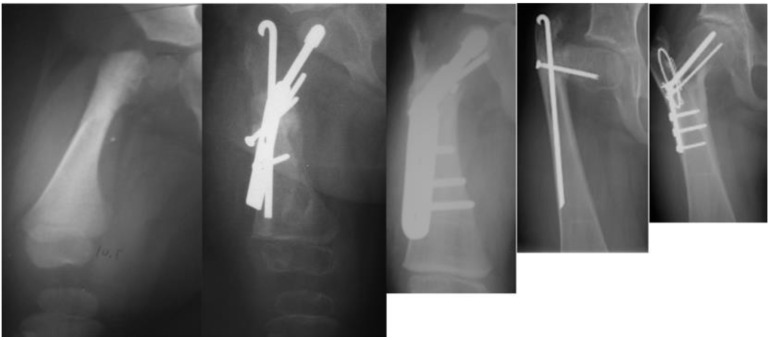
Radiographs showing: preoperative type 1b CFD (leftmost); after SUPERhip procedure fixed with infant sliding hip screw (second to left); the neck did not ossify so the infant sliding hip screw was changed to a larger size sliding hip screw (revision SH) a year later (middle); delayed ossification of the neck persisted and two years later the hip screw was removed and recurrent varus deformity occurred (second to right); a second revision SH was performed with blade plate fixation and insertion of BMP2 and the neck fully ossified (rightmost).

The occurrence of recurrent coxa vara and persistent delayed ossification were specifically analyzed (Figure 5). Statistical analysis was performed using Fisher’s exact test to compare the outcomes of the different treatment groups. All results are reported according to the two-tail *p*-value calculation.

## 3. Results

In the type 1b CFD primary SH surgery group, there were 35 males and 37 females. In the revision surgery group, there were 12 males and 14 females. The mean age at the time of primary SH surgery was 3.6 years (16 months–23.5 years). At the time of data review, the mean follow-up was 5.5 years (6 months–12.8 years). There were 25 cases of delayed ossification (35%), and 26 cases of recurrent varus (36%) (Table 1). All but one case of varus also had persistent delayed ossification. 

The 72 primary SH were divided into 3 groups: (1) non-fixed angle + no BMP2 (*n* = 34); (2) fixed angle + no BMP2 (*n* = 11); and (3) fixed angle + BMP2 (*n* = 27). The incidence of persistent delayed ossification of the femoral neck and recurrent varus were compared between each group.

The non-fixed angle + no BMP2 vs. fixed angle + no BMP2 groups were compared (Table 2). There was no statistically significant difference found between the occurrence of persistent delayed ossification between the two groups (*p* = 0.72). There was a statistically significant reduction in recurrent varus deformity in the fixed-angle group (*p* = 0.027). 

**Figure 6:**
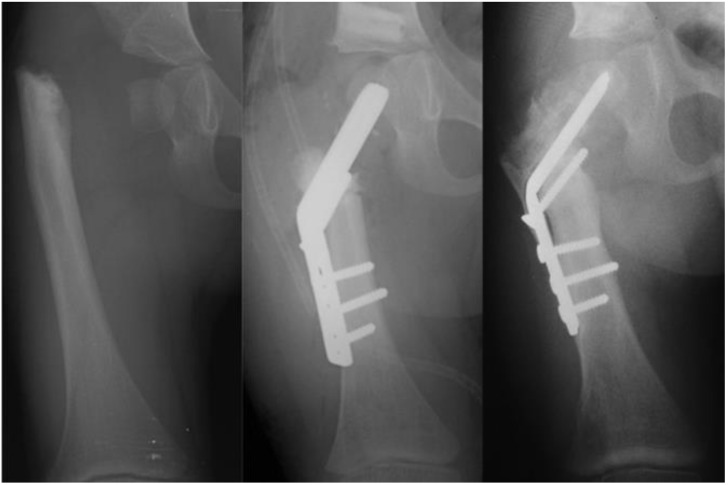
Type 1b CFD preop (left); immediately after SH procedure with fixed angle device and insertion of BMP2 into superior femoral neck (middle); and three months after SH procedure showing ossification of the superior femoral neck in the region of the BMP2 (right).

The fixed angle + no BMP2 vs. fixed angle + BMP2 groups were compared (Table 3). There was a significant reduction in persistent delayed ossification in the group treated with BMP2 (*p* = 0.045), but no significant difference was found in the occurrence of recurrent varus deformity between the two groups (*p* = 1.00) (Figure 6).

The non-fixed angle + no BMP2 vs. fixed angle + BMP2 groups were also compared (Table 4). There was a statistically significant reduction in both persistent delayed ossification (*p* = 0.0009) and recurrent varus deformity (*p* = 0.0002) in the fixed angle + BMP2 group. This is consistent with the previous group comparisons.

The 34 revision SH cases were also divided into 3 groups: (1) non-fixed angle + no BMP2 (*n* = 7); (2) fixed angle + no BMP2 (*n* = 17); and (3) fixed angle + BMP2 (*n* = 10). 

Of the 7 revision cases in the non-fixed angle + no BMP2 group, 3 (42%) had recurrent varus and 1 (14%) had persistent delayed ossification. There were 27 revision cases treated with fixed angle devices (10 +BMP2 and 17 no BMP2). When the fixed angle groups were compared, there was a significant decrease in the number of delayed ossification cases (*p* = 0.042) but no significant decrease in the number of recurrent varus deformity cases (*p* = 0.40) in the group that was treated with BMP2 (Table 5).

This finding that BMP2 insertion into the femoral neck leads to reduction in delayed ossification is consistent with the findings in the primary SH group. It is also not surprising that adding BMP2 to a fixed angle device does not reduce the incidence of recurrent varus as the fixed angle device prevents mechanical resistance to varus. 

The revision SH cases were combined with the primary SH cases to analyze the effect of BMP2. There were a total of 106 SH procedures performed on type 1b_2_ hips (72 primary + 34 revision). These again were divided into 3 groups: (1) non-fixed angle + no BMP2 (*n* = 41); (2) fixed angle + no BMP2 (*n* = 27); and (3) fixed angle + BMP2 (*n* = 38).

All 68 cases treated without BMP2 (group 1 + group 2) were compared with the 38 cases treated with BMP2 (group 3). The incidence of delayed ossification decreased from 42% to 13%, respectively (*p* = 0.0034). The incidence of recurrent varus decreased from 37% to 11% (*p* = 0.004) (Table 6). Of note, all cases with BMP2 were also treated with a fixed angle device. 

The combined patients treated with fixed angle + no BMP2 were compared with the patients treated with fixed angle + BMP2. There was a statistically significant reduction in the incidence of persistent delayed ossification (*p* = 0.018) but no significant difference in occurrence of recurrent varus deformity (*p* = 0.178). The combined patients treated with non-fixed angle + no BMP2 were compared with the group treated with fixed angle + BMP2. There was a statistically significant reduction in both persistent delayed ossification and recurrent varus in the group treated with a fixed angle device + BMP2 (*p* = 0.001 and *p* = 0.006, respectively).

## 4. Discussion

Congenital femoral deficiency (CFD) presents with a progressive spectrum of femoral deformity, proximal femoral deficiency, and femoral length discrepancy [1]. CFD was formerly referred to as proximal femoral focal deficiency (PFFD). The femoral deficiency ranges from complete absence of the hip and upper femur, to a pseudarthrosis of the femoral neck, to non-ossified cartilaginous segments of the proximal femur. Radiographic based classifications of PFFD often over-classified the degree of deficiency due to the lack of ossification of the proximal femur [17]. Paley referred to this as delayed ossification in his classification of CFD [1]. Boden et al. demonstrated severely disordered development of the proximal femoral physis in a 21-week fetus with CFD [18]. The cartilaginous anlage appeared normal, however the histopathology demonstrated failure of the physis to migrate proximally, as well as a lack of normal cellular organization. This inability of the physis to gain proper organization inhibits the endochondral ossification of the cartilaginous femoral neck, resulting in the inability to convert the cartilage to bone. The degree of disorganization likely correlates with the delay in ossification of the femoral neck that we observe in Paley type 1b cases. 

The secondary ossification center of the femoral head normally appears between four and six months after birth [19]. In other pediatric orthopedic conditions with delayed ossification, such as hip dysplasia, ossification of the femoral head is delayed. With restoration of hip stability and loading, ossification catches up. It is a reasonable assumption, then, that restoration of normal anatomy and biomechanics in type 1b CFD would lead to ossification of the femoral neck and subtrochanteric region. 

The first step was to understand the pathoanatomy of the upper femoral deformity. This was referred to only as coxa vara [20]. This is a gross oversimplification of a three-dimensional (3D) deformity. The actual deformity has both multiplanar bony deformities of the upper femur combined with corresponding contractures of the hip joint. Furthermore, these deformities are often of large magnitude, often reaching 90°. Paley deciphered the pathoanatomy by creating a model of the proximal femur position [2]. The proximal femur is normally shaped but moves into flexion, abduction, and external rotation. If one disconnects the distal femur from the proximal femur at the subtrochanteric level and reconnects it to the proximal femur in its flexed, abducted, retroverted position, relative to the pelvis, then the distal femur lies in neutral alignment with external rotation. Due to the proximal femur position, the soft tissues connected to it are contracted in flexion, abduction, and external rotation. To correct this pathoanatomical position of the femur, one needs to not only perform an osteotomy of the femur, but also soft tissue releases to free the proximal femur to return to its neutral anatomic position. Soft tissue releases of the flexors (tensor fascia lata, psoas, and rectus femoris), abductors (gluteus medius and minimus) and external rotator (piriformis) are combined with a subtrochanteric osteotomy of valgus, extension, and internal rotation. These are the elements that comprise the SUPERhip procedure which was developed in 1997 by the senior author (D.P.) (SUPER is an acronym for Systematic Utilitarian Procedure for Extremity Reconstruction) [2,3,4,5,6,7,8,9,10,11]. Initially, the osteotomy was fixed by inserting a Rush rod through the piriformis fossa down the femur and compressing the osteotomy using a lateral tension band cerclage wire around the Rush rod. This is referred to in this study as a non-fixed angle device. The initial procedure achieved a full correction of all of the deformities, restoring the neck shaft angle to normal. The senior author posited that the neck would ossify due to the normalization of the anatomy and biomechanics of the hip joint and the improved loading of the hip. Despite excellent surgical correction, the varus deformity recurred in a larger percentage of cases that had delayed ossification of the femoral neck. The senior author then switched to using a fixed angle device (sliding hip screw or blade plate), hoping this would prevent the recurrent varus from giving the femoral neck a chance to ossify under load. Although the incidence of recurrent varus seemed to decrease, the femoral neck failed to ossify in many cases. This was very disappointing given that the SUPERhip procedure was excellent at acutely correcting the severe deformity and pathoanatomy, only to watch the deformity either gradually recur or the neck remain unossified. As the logical next step, the senior author inserted BMP2 into the unossified cartilage, which often resulted in ossification within three months after insertion. The evolution of methodology occurred over the course of 10 years and was based on efforts to improve the results of the SUPERhip procedure. The target result is to prevent recurrent varus deformity of the proximal femur and to achieve ossification of the cartilaginous femoral neck. This evolution of treatment allowed us to evaluate two variables that may affect the achievement of these goals: non-fixed angle device vs. fixed angle device, with BMP2 vs. without BMP2. 

As expected, the use of a fixed angle device was better for preventing recurrent coxa vara. In addition, as was posited, inserting BMP2 into the femoral neck induced ossification. Using only a fixed angle device and not inserting BMP2 was less likely to lead to ossification of the femoral neck. BMP2 is considered off-label for use in children, but nevertheless is used for various indications in children, including congenital pseudarthrosis of the tibia. On the basis of experience in type 1b CFD, the senior author has also inserted BMP2 into the non-ossified cartilage of the tibial hemimelia anlage, which leads to ossification [21,22]. The senior author has also used BMP2 to weld two cartilage surfaces together when performing patelloplasty (Weber procedure) in order to fuse the patella to the head of the fibula when both are unossified [21,22]. Clearly, BMP2 is very potent when inserted into immature cartilage. The cellular mechanism of BMP2 appears to be based on the upregulation of endochondral bone healing. BMPs are important signaling molecules in normal human skeletal development, as well as in fracture repair. Endogenous BMP2 recruits mesenchymal stem cells from the surrounding vascularized muscle and periosteum and induces these pluripotent cells to differentiate into osteoblasts, thereby initiating the bone healing pathway [13,14]. The use of exogenous BMP2 mimics the normal cascade that occurs in endochondral bone maturation and fracture healing by stimulating the hyper-physiologic recruitment of osteoblastic progenitor cells [13,14]. In the setting of a cartilaginous femoral neck, this recruitment and cellular differentiation induces the existing cartilaginous anlage to convert rapidly to bone. The use of BMP2 has not been FDA cleared for this particular purpose nor evaluated for its use in children, and as such it is used in an off-label fashion. The use of BMP2 does carry a theoretical risk of oncogenesis, particularly in pediatric patients, due to mesenchymal stem cell transformation [13]. Parents/guardians were made aware of the risk and the off-label usage and informed consent was obtained prior to treatment. The recent literature, however, indicates that this risk may be lower than previously thought. In patients treated with BMP2 for spinal fusion, the risk of cancer was no greater than patients not treated with BMP2 [15]. No incidence of malignancy has been seen in this patient cohort, nor in any patient treated with BMP2 for other conditions by the senior author, over up to 14 years of follow-up [16]. Further follow-up of these patients will be needed to ensure long-term safety.

## 5. Conclusions

The SUPERhip procedure is extremely effective at the acute correction of the deformities associated with CFD type 1b. In the setting of a non-ossified femoral neck, the addition of a fixed angle device and BMP2 to the SH procedure has significantly decreased the rates of recurrent varus and persistent delayed ossification. The biomechanical and physiologic properties of these additions have led to them becoming a staple of the current SH procedure. Further follow-up of this patient cohort is needed to elucidate the long-term outcomes.

## Figures and Tables

**Table 1:** Total number of cases of delayed ossification and recurrent varus in primary SH for type 1b_2_ CFD. (CFD-Congenital Femoral Deficiency, SH-SUPERhip).

Late Complication	No. = 72	%
Delayed ossification	25	35
Recurrent varus	26	36

**Table 2:** Comparison of complications in primary SH: non-fixed angle + no BMP2 (group 1) vs. fixed angle + no BMP2 (group 2).

Complication	⦸Fixed < ⦸BMP		Fixed < ⦸BMP		*p*-Value
	No. = 34	%	No. = 11	%	2-tail
Delayed ossification	18	53	4	36	0.72
Recurrent varus	18	53	1	9	0.027

(BMP—Bone Morphogenic Protein, ⦸Fixed <—not fixed angle, ⦸BMP—no BMP, Fixed <—fixed angle).

**Table 3:** Comparison of complications in primary SH: Fixed angle + no BMP2 (group 2) vs. Fixed angle + BMP2 (group 3).

Complication	Fixed < ⦸BMP		Fixed < +BMP		*p*-Value
	No. = 11	%	No. = 27	%	2-tail
Delayed ossification	4	36	1	4	0.045
Recurrent varus	1	9	2	7	1.00

(Fixed <—fixed angle, ⦸BMP—no BMP, +BMP—plus BMP).

**Table 4:** Comparison of complications in primary SH: non-fixed angle + no BMP2 (group 1) vs. fixed angle + BMP2 (group 3).

Complication	⦸Fixed < ⦸BMP		Fixed < +BMP		*p*-Value
	No. = 34	%	No. = 27	%	2-tail
Delayed ossification	18	53	1	4	0.0009
Recurrent varus	18	53	2	7	0.0002

(⦸Fixed <—not fixed angle, ⦸BMP—no BMP, Fixed <—fixed angle, +BMP—plus BMP).

**Table 5:** Comparison of complications in revision SH: fixed angle + no BMP2 (group 2) vs. fixed angle + BMP (group 3).

Complication	Fixed < ⦸BMP		Fixed < +BMP		*p*-Value
	No. = 17	%	No. = 10	%	2-tail
Delayed ossification	8	47	1	10	0.042
Recurrent varus	6	35	2	20	0.405

(⦸BMP—no BMP, Fixed <—fixed angle, +BMP—plus BMP).

**Table 6:** Comparison of complications in all SH (primary + revision): no BMP (groups 1 and 2) vs. + BMP (group 3).

Complication	⦸BMP		+BMP		*p*-Value
	No. = 68	%	No. = 38	%	2-tail
Delayed ossification	28	41	5	13	0.0034
Recurrent varus	25	37	4	11	0.0040

(⦸BMP—no BMP, +BMP—plus BMP).
